# Goblet cell carcinoid of the rectum: a case report

**DOI:** 10.1186/s40792-020-00937-3

**Published:** 2020-07-18

**Authors:** Yoshiyuki Inoue, Hisanaga Horie, Yuko Homma, Ai Sadatomo, Makiko Tahara, Koji Koinuma, Hironori Yamaguchi, Toshiki Mimura, Atsushi Kihara, Alan Kawarai Lefor, Naohiro Sata

**Affiliations:** 1 Department of Surgery, Division of Gastroenterological, General and Transplant Surgery, Jichi Medical University, 3311-1 Yakushiji Shimotsuke, Tochigi, 329-0498 Japan; 2 Department of Pathology, Jichi Medical University, 3311-1 Yakushiji Shimotsuke, Tochigi, 329-0498 Japan

**Keywords:** Goblet cell carcinoid, Rectum, Liver metastases

## Abstract

**Background:**

Goblet cell carcinoid (GCC) is a neuroendocrine tumor usually found in the appendix. GCCs exhibit characteristic findings with mixed endocrine-exocrine features such as staining positive for neuroendocrine markers and producing mucin. The primary GCC of the rectum is exceedingly rare.

**Case presentation:**

A 77-year-old Japanese male presented with hematochezia. Anal tenderness and a hard mass in the anal canal were found on the digital rectal examination, and colonoscopy was performed. Colonoscopy showed an irregularly shaped mass in the anal canal. Biopsy showed mixed features including adenocarcinoma in situ, well-differentiated adenocarcinoma, and mucinous carcinoma with invasive proliferation. No metastatic lesions were found on the computed tomography scan. Pelvic magnetic resonance imaging scan showed extramural growth of a tumor on the ventral side of the rectum without invasion to the prostate. Laparoscopic abdominoperineal resection was performed. The final diagnosis was well-differentiated adenocarcinoma in the mucosa and goblet cell carcinoid from the submucosa to the adventitia of the rectum. The patient was discharged from the hospital on postoperative day 16. Six months after resection, a computed tomography scan revealed multiple metastatic lesions in the liver. Several chemotherapy regimens were given, and the patient has stable disease 27 months after surgery.

**Conclusion:**

We present a patient with rectal GCC with metachronous liver metastases. Since GCC grows intramurally and is biologically aggressive compared to typical carcinoid lesions, the disease is usually diagnosed at an advanced stage. The development of optimal adjuvant chemotherapy is needed for those patients.

## Background

Neuroendocrine tumors (NET) are much less common than adenocarcinomas and may occur in almost any organ [1]. The majority of NETs are found in the gastrointestinal tract, pancreas, and bronchopulmonary system [2]. Goblet cell carcinoid (GCC) is a NET tumor in the World Health Organization classification and is found mainly in the appendix [3]. The primary GCC of the rectum is exceedingly rare [3]. GCC exhibits characteristic findings with mixed endocrine-exocrine features such as staining positive for neuroendocrine markers and producing mucin. GCCs are biologically aggressive lesions, more similar to adenocarcinomas than typical carcinoid tumors, also a type of NET [4]. We describe a patient with an advanced rectal GCC treated with radical resection and chemotherapy for metachronous liver metastases.

## Case presentation

**Fig. 1:**
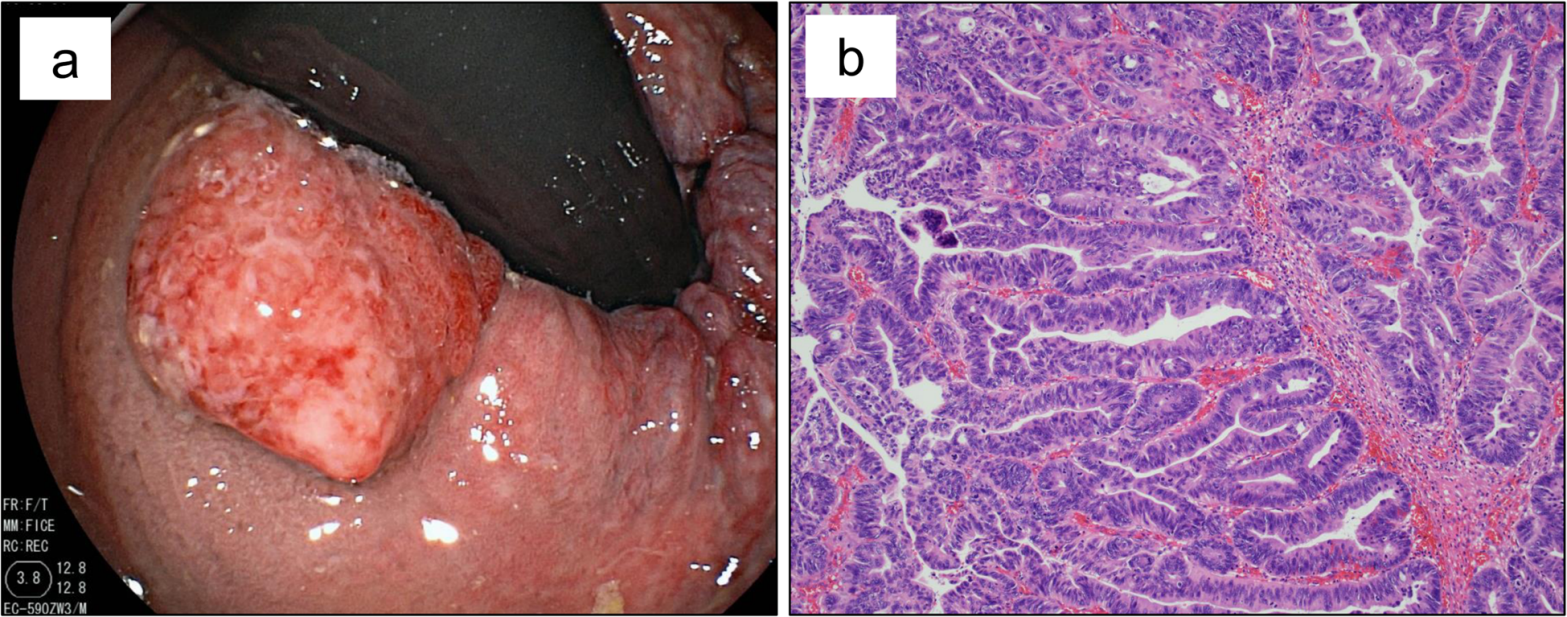
Colonoscopy 5 years prior to presentation revealed a 13-mm semi-pedunculated polyp in the rectum. **a** Histologic findings showed well-differentiated adenocarcinoma in situ. **b** (×200)

**Fig. 2:**
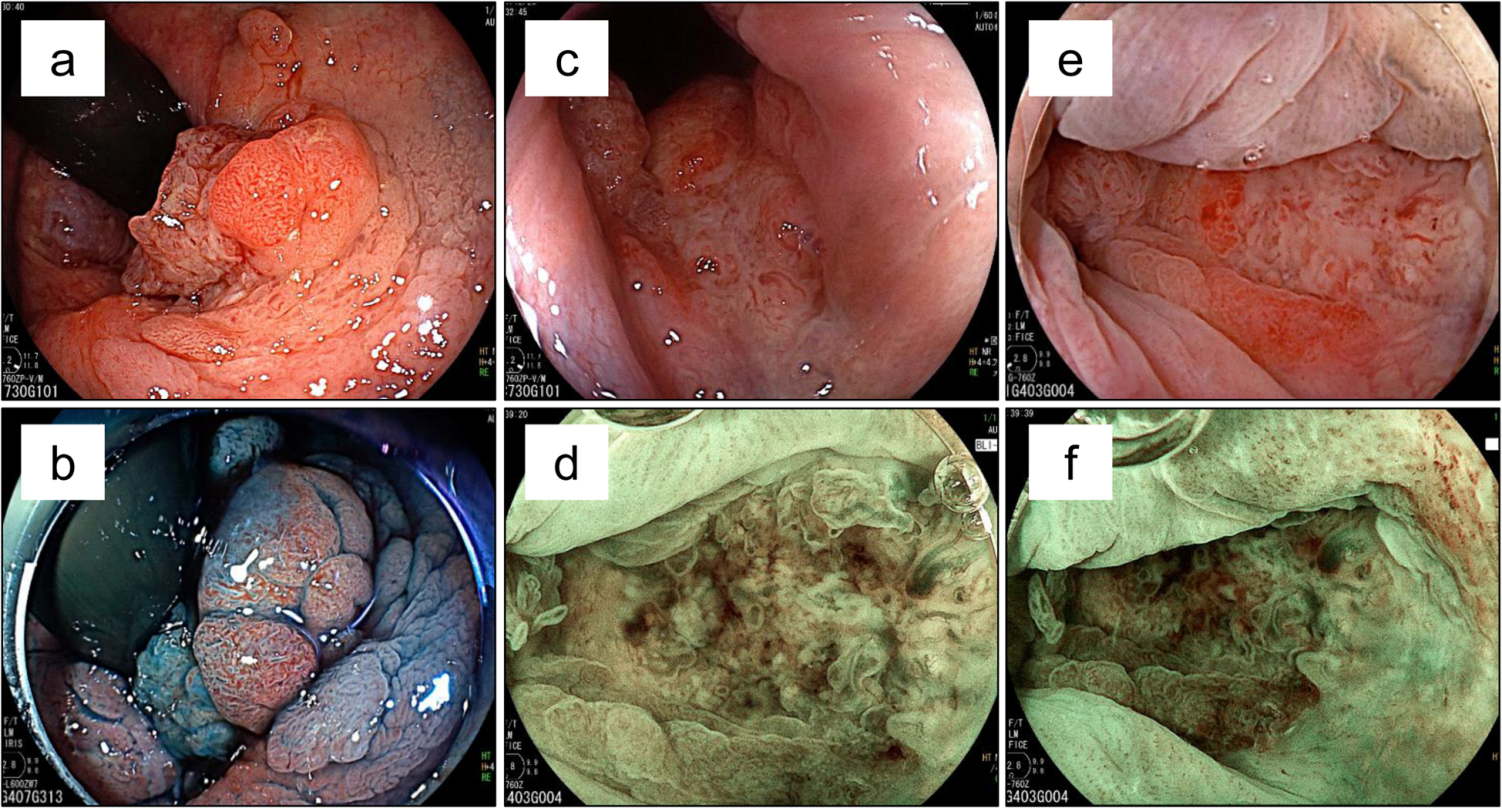
Colonoscopy revealed an irregular shaped mass from the proximal anal canal to the anal verge

**Fig. 3:**
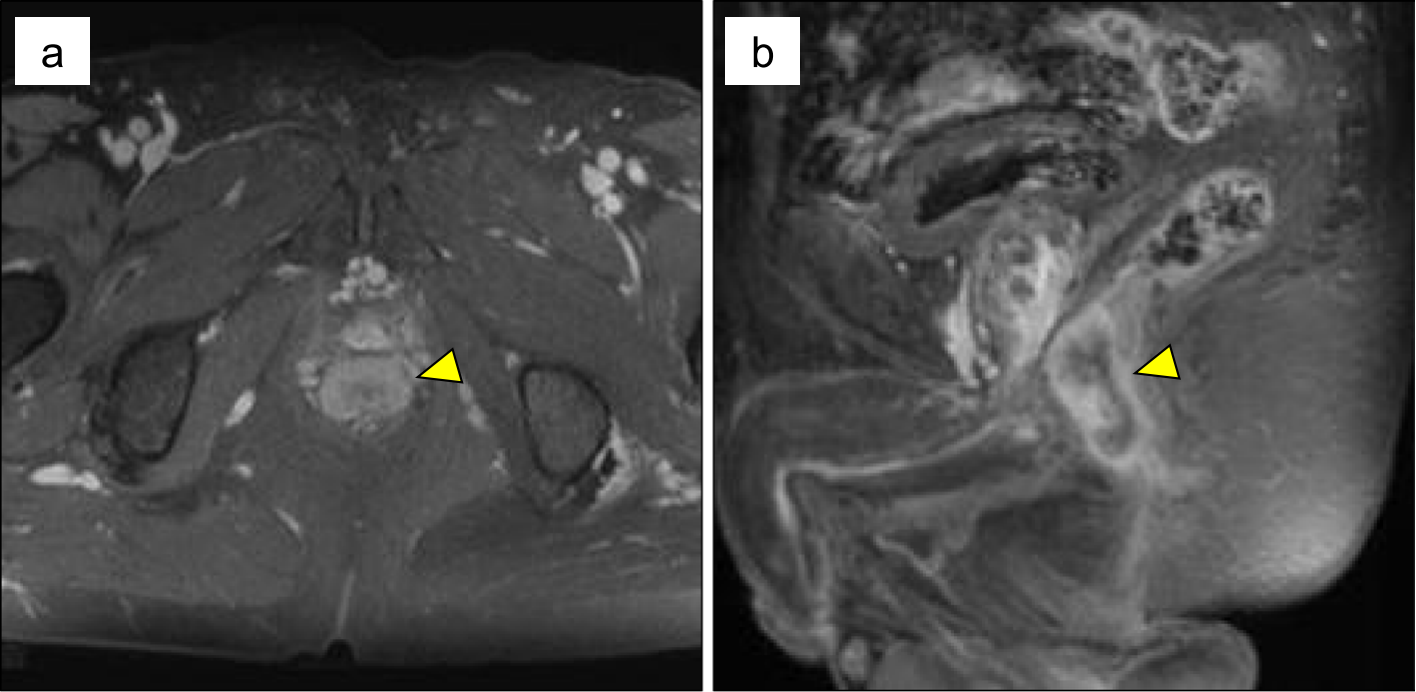
Pelvic magnetic resonance imaging scan demonstrated the extramural growth of the tumor on the ventral side of the rectum. There was no invasion of the prostate. **a** Axial image. **b** Sagittal image

A 77-year-old Japanese male presented with hematochezia. Five years prior to presentation, he underwent trans-anal mucosal resection of a 13-mm semi-pedunculated polyp of the rectum. Histologic findings showed well-differentiated adenocarcinoma in situ; however, the horizontal margin was unclear due to cauterization of the lateral edge of the tumor (Fig. 1). After resection, magnified endoscopy was performed and showed no atypical pit pattern. The patient takes Amlodipine for hypertension. The patient had a history of hypertension and no allergies. He drank distilled spirits daily for 57 years. He smoked one and a half pack of cigarettes for 20 years and quit smoking 37 years ago. His father died of esophageal carcinoma. Anal tenderness and a hard mass in the anal canal were found on the digital rectal examination. The remainder of the physical examination was unremarkable. Laboratory tests showed: Hb 13.4 g/dl, CEA 2.4 ng/ml (normal value < 4.5), and CA19-9 10 U/ml (normal value < 36). Colonoscopy revealed an irregular mass in the anal canal with three distinct areas. A sessile polyp was located in the proximal anal canal (near the anorectal line) (Fig. 2a, b), a villous-appearing lesion in the middle of the anal canal (near the dentate line) (Fig. 2c, d) and a depressed hard lesion from the inferior part of the anal canal to the anal verge (Fig. 2e, f). Endoscopic biopsy showed adenocarcinoma in situ, well-differentiated adenocarcinoma, and mucinous carcinoma with invasive proliferation at the site of each lesion. No metastatic lesions were found on computed tomography (CT) scan. Pelvic magnetic resonance imaging scan showed extramural growth of a tumor on the ventral side of the rectum; however, there was no invasion to the prostate (Fig. 3).

**Fig. 4:**
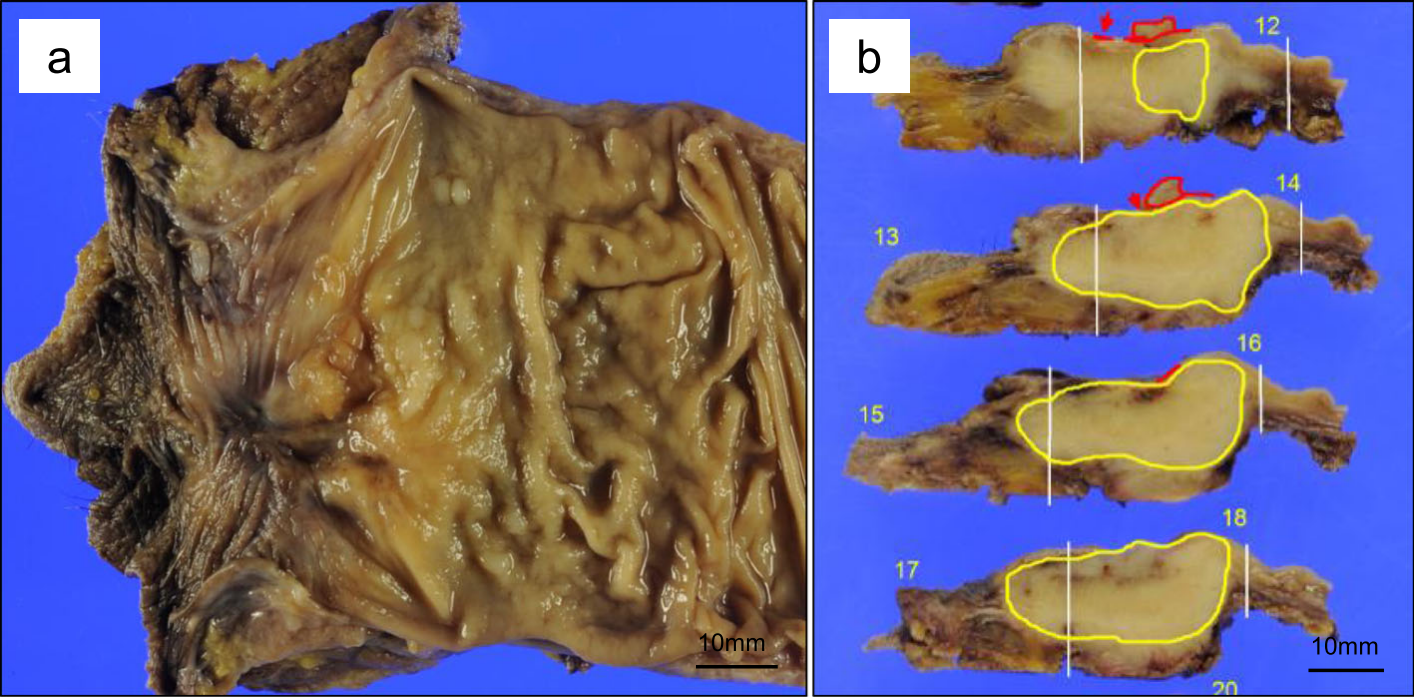
Depressed and protruding lesions were found from the proximal anal canal to the anal verge macroscopically. **a** The surface was a gray-white mass from the submucosa to the adventitia. **b** Area surrounded by the red line delineates the well-differentiated adenocarcinoma component the yellow line delineates the goblet cell carcinoid component

**Fig. 5:**
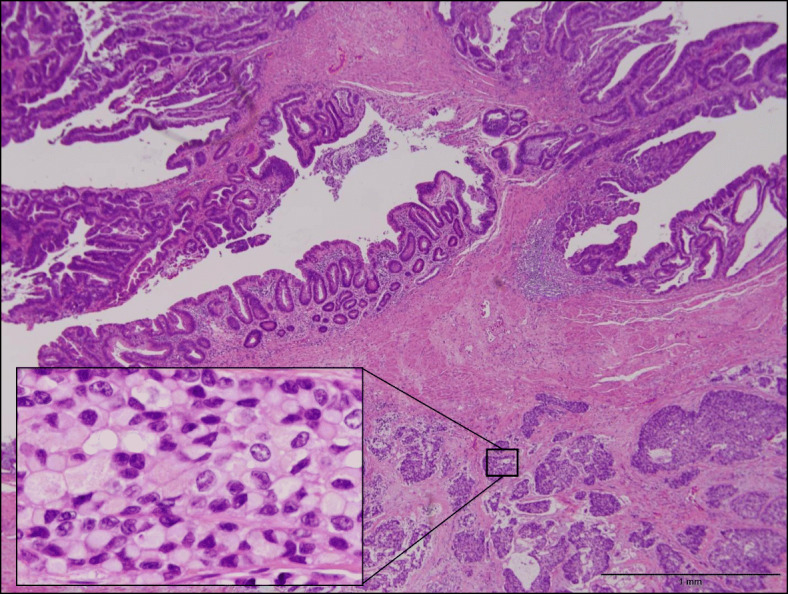
Histopathologic evaluation showed a malignant neoplasm with goblet-like cells. Malignant cells with nests were found from the submucosa to the adventitia with lymphatic and venous invasion. Components of well-differentiated adenocarcinoma were also found in the mucosa (hematoxylin and eosin ×10, a small window shows a magnified image of goblet-like cell components ×400)

**Fig. 6:**
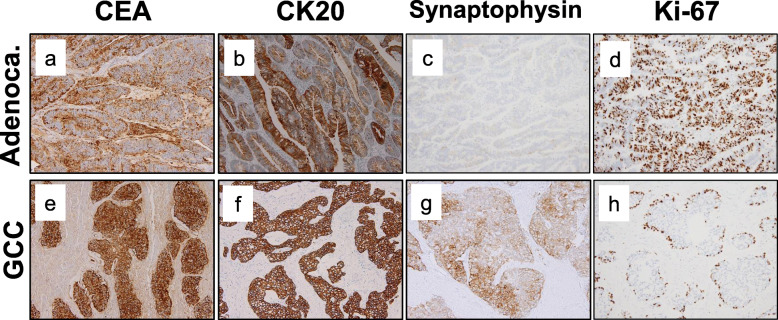
Adenocarcinoma components stained positive for CEA (**a**), CK20 (**b**) as epithelial marker, and negative for synaptophysin (**c**). Goblet-like cells stained positive for CEA (**e**) and CK20 (**f**) as epithelial markers, synaptophysin (**g**) as a neuroendocrine marker by immunohistochemistry. Ki-67 had a different pattern from the adenocarcinoma components (**d**) and GCC (**h**) being diffuse and peripheral in the nests for each area respectively (×20)

The lesion was felt to be resectable and laparoscopic abdominoperineal resection was performed. Intraoperatively, the tumor was found tightly adherent to the dorsal surface of the prostate, necessitating the removal of a portion of the prostate. The tumor measured 3.0 × 3.5 × 1.5 cm (Fig. 4). Histopathologic evaluation showed a malignant neoplasm with goblet-like cells. Malignant cells with nest formation were found from the submucosa to the adventitia and invaded into the anal verge. Components of well-differentiated adenocarcinoma were also found in the mucosa. These goblet-like cells showed lymphatic invasion, venous invasion, and perineural invasion (Fig. 5); however, a well-differentiated component did not show any microscopic invasive features. The surgical resection margins were negative for malignant cells. Goblet-like cells stained positive for CK7, CK20, CAM5.2, synaptophysin, CD56 (weak), chromogranin A (a few), CDX-2, Ki-67 (only peripheral areas in the nests), CEA, MUC2, MUC5AC(a few), and negative for serotonin and somatostatin by immunohistochemistry. Ras and BRAF were wild types. Adenocarcinoma components stained positive for CK20, CDX-2, Ki-67, and CEA (Fig. 6). The final diagnosis was well-differentiated adenocarcinoma in the mucosa and GCC from the submucosa to the adventitia of the rectum. The patient was discharged from the hospital on postoperative day 16.

**Fig. 7:**
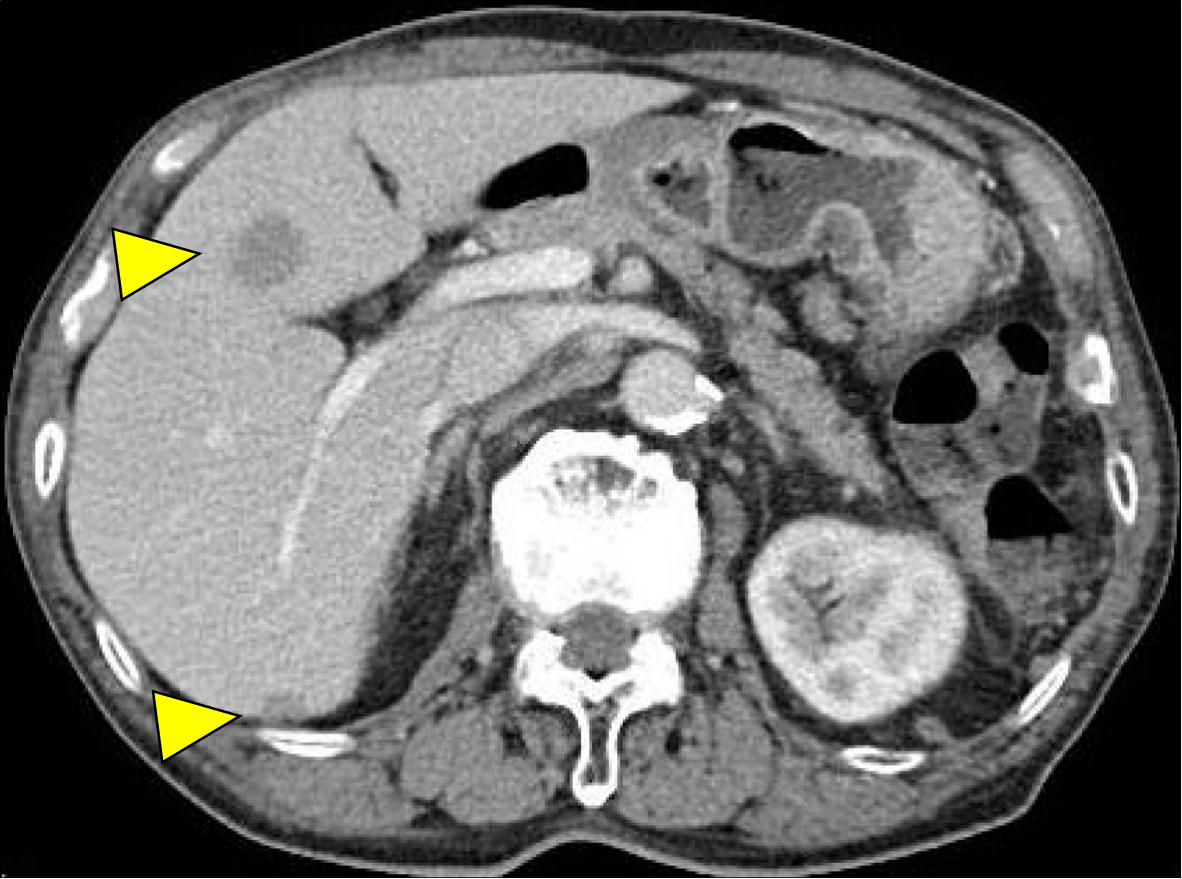
Abdominal computed tomography scan 6 months after surgery revealed multiple metastatic lesions in the liver. A total of 5 lesions were found

Six months postoperatively, a CT scan revealed multiple metastatic lesions in the liver (Fig. 7). Although there is no established chemotherapy regimen for goblet cell carcinoid, the patient was initially treated with 4 courses of 5-fluorouracil, leucovorin, oxaliplatin (FOLFOX), and bevacizumab [5]. As a second regimen, capecitabine + oxaliplatin + bevacizumab (CapeOX) were selected. After 17 courses of CapeOX, the progression of the liver metastases was noted. As a third regimen, irinotecan plus oral S-1 (a combination of tegafur, 5-chloro-2, 4-dihydroxypyridine, and potassium oxonate) + bevacizumab was used, and 5 courses have been given to date. The liver disease is now stable, 27 months postoperatively.

## Discussion

The primary GCC of the rectum is a rare lesion [3]. GCC was first described by Gagne in 1969 [3] and is known by many names including adenocarcinoid, mucinous carcinoid, microglandular GCC, amphicrine neoplasm, mucin-producing neuroendocrine tumor, or carcinoma and crypt cell carcinoma because this lesion has unique mixed endocrine-exocrine features [6]. Multiple names for this lesion make it difficult to review the literature and understand the character of this tumor. Carcinoid-related tumors are classified as carcinoid (well-differentiated neuroendocrine neoplasm) and mixed endocrine–exocrine carcinoma by the World Health Organization histological classification of tumors of the appendix. The latter includes tubular carcinoid, GCC (mucinous carcinoid), and mixed carcinoid adenocarcinoma [7]. Höfler et al. reported that GCCs are derived from undifferentiated stem cells and are different from typical carcinoids that originate from enterochromaffin cells (Kulchitsky) in the mucosal stroma [8].

Subbuswamy et al. described the first large series of GCC in 1974 [9] and found that the appendix is the most common site for these tumors. Other sites, such as the stomach, duodenum, small intestine, biliary tract, and bronchus, have also been reported [3, 10, 11]. It is rarely found in the lower gastrointestinal tract. The incidence of GCC is 1.2/1000000 people per year among Caucasian women [12]. Appendiceal GCC is found in 0.3–0.9% of appendectomy specimens [6], and approximately 14–19% of primary appendiceal cancers [12]. The mean age at diagnosis is 58 years, and there is no significant difference in incidence between males and females [12]. No specific risk factors have been identified to date [12]. Compared to appendiceal GCC, rectal GCC is much less common, and its etiology is unknown. Patients reported in the literature to date with GCC are summarized in Table 1. Zhang et al. reported that the characteristic pathological findings of GCC usually include concentric infiltration of the appendiceal wall by small tight clusters, nests, or cords of tumor cells. GCCs exhibit a goblet cell morphology with a small compressed nucleus and rich intracytoplasmic mucin [6]. Immunohistochemistry stains are positive for neuroendocrine markers such as synaptophysin, chromogranin, CD56, and positive for epithelial markers such as CEA, CK7, CK20, and CDX2. MUC2 is also positive as a secretary mucin marker derived from intestinal goblet cell [3, 12].

**Table 1:** Patients previously reported with goblet cell carcinoid in the rectum

No.	Author	Year	Age	M/F	Coexisting component	Tumor depth	Synchronous metastasis	Treatment	Adjuvant chemotherapy	Site of recurrence	Treatment of recurrence	Prognosis (months)
1	Ishii [13]	2001	71	M	Typical carcinoid, WDA	T4b	Regional lymph node	Miles’ operation	None	Subcutaneous, testis	None	Died (9)
2	Kato [14]	2002	44	M	None	T3	None	Total colectomy (accompanied by UC)	None	Liver, brain, peritoneum	Right lobectomy of the liver, intraperitoneal administration of CDDP	Died (39)
3	Wakahara [15]	2010	58	M	Typical carcinoid	T4b	Pelvic lymph node	Total pelvic exenteration, LLND	5-FU, LV	Groin and pelvic lymph node	Lymphadenectomy	Alive (> 60)
4	Yamabuki [7]	2011	75	M	None	T2	Regional lymp node	Miles’ operation	None	Lung	FOLFOX	Alive (15)
5	Kang[16]	2016	46	F	None	T1	None	Low anterior resection	None	None	None	Alive (10)
6	Present patient	2020	77	M	Wda	T3	None	Miles’ operation	None	Liver	FOLFOX+BV, CapeOX+BV, IRIS+BV	Alive (27)

*WDA* well-differenciated adenocarcinoma; *n.d.* not described; *UC* ulcerative colitis; *LLND* lateral lymph node dissection; *CDDP* cisplatin, 5-FU; *LV* fluorouracil and leucovorin; *FOLFOX* 5-fluorouracil, leucovorin, oxaliplatin; *BV* bevacizumab; *CapeOX* capecitabine + oxaliplatin; *IRIS* irinotecan plus oral S-1 (a combination of tegafur, 5-chloro-2, 4-dihydroxypyridine, and potassium oxonate); *M* male; *F* female

In the present patient, the GCC lesion was positive for epithelial markers and neuroendocrine markers supporting the presence of dual endocrine-exocrine features. Lymphatic and vascular invasion were only found in the GCC component of the lesion while the well-differentiated component grew in situ. Metastatic lesions in the liver would usually be thought to be derived from the GCC component. Surgical resection has been recommended for rectal GCC due to its aggressive features [15]. In recent years, the coexistence of a high-grade adenocarcinoma with GCC has been called adenocarcinoma ex GCC or mixed GCC-adenocarcinoma and affects the prognosis [6]. The adenocarcinoma component seems to be responsible for the biologic aggressiveness of the tumor [6]. Burke et al. reported that of 10 patients with mixed GCC-adenocarcinoma lesions, 8 patients died of metastatic carcinoma, 1 remained alive with disease, and 1 alive without disease while among 25 patients with GCC only lesions, there were no patients with metastatic lesions or who died of disease [17]. Another report classified 4 groups based on the volume of the adenocarcinoma component as follows: Group 1—GCC or GCC with < 25% adenocarcinoma; group 2—GCC with 25–50% adenocarcinoma; group 3—GCC with > 50% adenocarcinoma; and group 4—adenocarcinoma without a GCC component). The overall survival was a mean (SD) of 83.8 (34.6), 60.6 (30.3), 45.6 (39.7), and 33.6 (27.6) months for each group respectively [18]. These two reports show that the coexistence of an adenocarcinoma component inside the GCC mass played a determining role in the prognosis of the patients. However, in the present patient, the adenocarcinoma lesion was located only in the mucosa and did not show aggressive behavior such as lymphatic or vascular invasion. Therefore, it is reasonable to think that the GCC grew and infiltrated from the rectum to the anal canal wall and resulted in a metastatic lesion in the liver after resection. It is unclear why the GCC component and the adenocarcinoma component are adjoining.

The present patient had a history of a trans-anal mucosal resection of a rectal in situ carcinoma, so there is a small chance that this represents tumor recurrence. Other mechanisms can be considered such as differentiation from adenocarcinoma to GCC or transformation from GCC to adenocarcinoma or bidirectional transformation from a common precursor [19, 20]. However, it is very difficult to distinguish these mechanisms morphologically. Zhang et al. recommend that treatment should be based on the tumor stage based on the staging of typical adenocarcinoma [6]. Gilmore et al. reported on the prognosis of appendiceal GCC [12]. They found a large difference in survival for stage I/II and stage III/IV disease (5-year overall survival 22% and 14%, respectively. Adjuvant chemotherapy should be given to patients with recurrence or distant metastases [15]. The rarity of GCC makes it difficult to perform a randomized controlled trial or establish clinical guidelines for treatment. There is little information regarding the optimal adjuvant treatment after resection. However, adjuvant 5-fluorouracil (5-FU)-based regimen is recommended for patients with stage III or stage IV disease according to the treatment of colon cancer [12, 15], and we observed the effectiveness of a 5-FU-based regimen in the present patient.

## Conclusion

GCC of the rectum is an extremely rare tumor composed of mixed malignant endocrine and exocrine cells. The present patient had a highly aggressive tumor with a malignant clinical course, despite the lack of an adenocarcinoma component in this GCC. Standardization of the classification system and development of optimal adjuvant chemotherapy may be important for the effective management of these patients with this rare disease.

## Data Availability

Not applicable

## Abbreviations

GCC: Goblet cell carcinoid
NET: Neuroendocrine tumor
FOLFOX: 5-Fluorouracil, leucovorin, oxaliplatin
CT: Computed tomography
